# “Humans and records are entangled”: empathic engagement and emotional response in archivists

**DOI:** 10.1007/s10502-022-09392-5

**Published:** 2022-05-09

**Authors:** Cheryl Regehr, Wendy Duff, Henria Aton, Christa Sato

**Affiliations:** 1 Factor-Inwentash Faculty of Social Work, University of Toronto, Toronto, Canada; 2 Faculty of Information, University of Toronto, Toronto, Canada

**Keywords:** Traumatic archives, Empathic engagement, Emotional empathy, Cognitive empathy, Radical empathy, Archivists

## Abstract

There is growing awareness in archival communities that working with records that contain evidence of human pain and suffering can result in unsettling emotions for archivists. One important finding of this work, however, is the considerable variability in not only the nature of responses, but also the nature of records that provoke emotional responses. Using in-depth qualitative interviews with 20 archivists from across Canada and one from the United States, and employing grounded theory methodology, this study sought to better understand the nature of emotional responses and factors associated with distress. Archivists described a wide range of reactions including shock, intrusive thoughts, profound senses of anger, sadness and despair, and ultimately at times disrupted functioning in personal and occupational spheres. One factor that has been associated with increasing vulnerability to distress in other occupational groups is empathic engagement, which is understood to have two elements: a vicarious emotional process and a cognitive process. This article explores the impact of personal connections and the nature of empathic engagement between archivists, donors, community researchers, and the records themselves on emotional response.

## Introduction

As “co-witnesses” to the lives and stories they archive, archivists and archival scholars have the potential to be deeply affected by records, especially by those containing emotionally challenging or sensitive accounts of human suffering and survival (Punzalan 2009). Fiona Murphy suggests that while we may primarily see the archive as a storehouse of memory and fact, it is in fact a repository of “trauma and pain, sorrow and loss for many, where unpacified ghosts with unfinished business await”(Murphy 2011, p481). Archival scholars, Anne Gilliland and Michelle Caswell have described this as “the capacity of records and archives to motivate, inspire, anger, and traumatize” (Gilliland and Caswell 2016, pp55–56). Recently, there has been a surge of academic and professional interest in the affective potential of archives (Cifor and Gillil 2016), in which as James Lowry notes, “the personal, embodied and emotional is given space and serious treatment”(Lowry 2019, p190). This has included a growing awareness of the impact of working with records with traumatic potentialities (Sexton 2019) on archivists themselves (Nathan et al. 2015; Sexton 2019; Sloan et al. 2019; Aton et al. in review).

Earlier authors described the impact on researchers of working with troubling archives. For instance sociologist Jo Moran-Ellis described a “pain by proxy” in response to working with records of child sexual abuse (Moran-Ellis 1997, p181). Other authors describe intrusive thoughts, disturbed sleep, and needing to talk about disturbing cases with unwilling relatives and friends as a result of reviewing suicide records in a coroner’s office (Fincham et al. 2008). More recently, in survey research, archivists identified emotional responses experienced when encountering certain types of records (Sloan et al. 2019; Aton et al. in review). One important finding of this work, however, is the considerable variability in not only the nature of responses, but also the nature of records that provoke these responses. Katie Sloan and colleagues note the difficulty in predicting what materials will be unsettling stating “Some records are not necessarily traumatic by definition but nevertheless evoke a traumatic response” (Sloan et al. 2019, p12). How might variability in responses of archivists to potentially distressing records be understood?

Significant research with other occupational groups has studied the factors that contribute to an individual’s susceptibility or resilience to distress when exposed to traumatic stimuli in the workplace. This has included factors that are specific to the individual, including previous trauma exposure, resilience, optimism and coping styles (Collins 2007), and the existence of a personal network of social supports (Regehr 2009; Marmar et al. 2006). Previous research has also included factors related to the traumatic exposure itself including: the content (Regehr et al. 2007); the form it takes (Polak et al. 2019), the intensity and length of exposure (Resnick et al. 1992), and the degree to which it is personally meaningful (Regehr et al. 2002). To date however, factors associated with emotional response specifically of archivists are largely unexplored. This article reports on a study that explored the impact of personal connections and the nature of empathic engagement between archivists, donors, community researchers, and the records themselves on archivists’ emotional responses. Using in-depth qualitative interviews with 21 archivists and employing grounded theory methodology, this study sought to better understand the nature of emotional responses and factors associated with distress.

### Empathic engagement as a theoretical framework

One factor that has been identified in other occupational fields as increasing vulnerability to stress and distress is empathic engagement with others (Regehr et al. 2002; Brockhouse et al. 2011). Philosophers, researchers and mental health professionals have long considered, debated and examined the concept of empathy, but this work can be largely summarized as comprising two main elements –affect and cognition (Regehr 2018; Davis 1996, 1983).

The first component of empathy is seen to be a vicarious emotional process in which the person develops an affective connection with another and subsequently has an emotional response to the other’s suffering (Hume 1777; Keefe 1976). In this conceptualization, through witnessing and attending to another’s emotional state, an individual is provoked to experience a similar, although weaker reaction (Davis 1996). One formulation regarding the roots of emotional empathy is biological, arising from the finding that fear induced through social observation engages similar neural mechanisms as direct personal experience with adverse events (Olsson et al. 2007). For instance, using MRIs Beatrice De Gelder and colleagues demonstrated that viewing bodily expressions of fear in others produces higher activity in areas of the brain known to process emotional information (De Gelder et al. 2004). Similarly, brain activity is stimulated when one imagines, anticipates or observes pain in others (Shirtcliff et al. 2009), provoking emotion-driven body-related changes “*as if*” the person had experienced the pain directly themselves (Damasio 2001). Several studies have demonstrated that affective and cognitive empathy emanate from different areas of the brain, confirming early theoretical models for understanding empathy as two distinct processes (Eres et al. 2015; Fan et al. 2011; Shamay-Tsoory et al. 2009).

Drawing on the work Joan Koss-Chioino (2006), Michelle Caswell and Marika Cifor (2016) suggest a more deliberate approach to emotional empathy in the form of “radical empathy”. Radical empathy is described as an intimate encounter in which individual experiences can be “melded into one field of feeling and experience” (Koss-Chinoino 2006). Caswell and Cifor (2016) contend “Our conception of empathy is radical in its openness and its call for a willingness to be affected, to be shaped by another’s experience, without blurring the lines between self and the other”(p31). Judith V. Jordon and Harriet L. Schwartz suggested that in order for radical empathy to create change, it must include a sense that the individual has been touched, impacted or influenced by the situation of another (Jordan and Schwartz 2018).

Caswell and Cifor nevertheless caution that such an approach presents “the possibility of grave danger for archives and archivists”(Caswell and Cifor 2016, p32). In this respect, archivists are warned to be careful not to “appropriate the experiences of others under the guise of empathy” (Caswell and Cifor 2016, p33). Further, emotional empathy can potentially lead to empathic strain on the part of professionals (Regehr et al. 2002; Brockhouse et al. 2011). This may include affective dysregulation in response to exposure to violent imagery or other disturbing material; retriggering experiences in the practitioner’s own history; depletion of energy; or lead to cognitive disillusionment in the world. John P. Wilson and Rhiannon B. Thomas suggest that unaddressed empathic strain can lead to prolonged disequilibrium, enmeshment, withdrawal, or empathic repression (Wilson and Thomas 2004).

The second component of empathy is a cognitive process in which an empathic individual can accurately imagine the viewpoint of others and perceive their plight. From this perspective, empathy can be seen to be an objective, analytical process (Kant 1788; Rogers 1957), the result of which allows an individual to behave in a manner that conveys concern and caring. Famous psychotherapist Carl Rogers, for instance, defined empathy as the ability to perceive the internal frame of reference of another with accuracy, as if one were that person but without ever losing the *“as if”* condition (Rogers 1957). Thus, the helper is able to consider the consequences of his/her actions on the welfare of others (Hogan 1969) and act in their best interests, without feeling their pain.

In summary, the two elements of empathy have implications for the development of stress and trauma reactions in professions focused on helping others (Regehr et al. 2002; Cadge and Hammonds 2012). Empathy may be expressed as a cognitive understanding of the distress of individuals with whom we work, while at the same time maintaining a degree of emotional distance. Alternately, it may involve an emotional connection with the individual in which their emotions are transmitted to the other. The latter form of empathy presumably increases vulnerability to symptoms of stress and trauma in the individual. That is, as a result of observing the embodied emotions of others, such as loneliness and despair, these same emotions may be mirrored in the individual in a sort of emotional contagion. Vittorio Gallese suggests that the crucial element is a meaningful relational link between the agent and the observer (Gallese 2001). Most emphatic research to date has focussed on connections or understanding of the distress of other living beings. However, archivists and scholars who use archival sources also make connections and gain understanding of people represented in record. Historian Penny Russell, as quoted in Brennan 2018, posits that “in ‘*being* in the archives reading letters, diaries and other personal material, seeking the contours of a life, the constructions of self, the moments of dramatic or intense emotion’, the researcher of historical subjects, in the ‘act of reading, can enter fleetingly into relationships of affect and empathy with those long-dead chroniclers of sorrow and joy, anger and embarrassment, pleasure and pain’ (Brennan 2018, p6).

### The current study

Arising from the results of a pilot study conducted by two authors of this paper, we believe that there is an urgent need to develop a more in-depth understanding of how archivists respond to their work, how different types of records and functions impact emotional responses, and the ways archival programs and institutions can support their students and employees. Using in-depth qualitative interviews, this article seeks to better understand the nature and factors associated with emotional responses in archivists as they work with records with traumatic potentialities (Sexton 2019), support researchers looking for information in records, or assist individuals who are donating their records to the archives.

## Methods

This project adopted a discovery-oriented qualitative approach, utilizing grounded theory method, originally developed by Barney Glaser and Strauss (1967). Kathy Charmaz (2014, 2000) proposed an adaptation to grounded theory, constructivist grounded theory (CGT) in which data and meaning are co-constructed through the relationship between the researchers and participants (Chun Tie et al. 2019; Birks and Mills 2015; Charmaz 2017). To this end, this research engaged in dialogues using the long-interview method of data collection (McCracken 1988) in support of thick description and credibility (Lietz and Zayas 2010). The interviews were conducted by two members of the research team: a doctoral student in Information Studies and experienced archivist; and a doctoral student in Social Work and experienced clinician. The proposal was approved by the Human Subjects Research Ethics Board at University of Toronto.

Twenty-one archivists participated in the semi-structured interviews; 20 interviews were conducted in English and one in French. Five archivists identified as male, fifteen as female, and one as non-binary. While most archivists self-identified as white with mixed backgrounds, others self-identified as Indigenous, Métis, Jewish, and Japanese (specific numbers are excluded to maintain confidentiality). One archivist identified as disabled. About half of the archivists had 6–10 years of archives work experience; five had 5 or fewer years of work experience in archives, and four had 20 + years of experience. Seventeen archivists had formal graduate training in archives or library science. Eight currently worked in archives connected to their community, three additional archivists had previously worked in archives connected to their community.

Interviews were transcribed verbatim for line by line micro-analysis and coding, allowing researchers to interact with the data and examine emerging themes. The two senior researchers (one a professor of social work and one a professor of archival science) directly engaged in the data analysis, coding transcripts to determine germane categories (Strauss and Corbin 1998). Emerging themes and patterns were identified and categorized, followed by determining inter-relationships in an iterative and reflexive manner (Ben-Ari and Enosh 2011).

## Results

The first set of themes arising from this analysis relates to *Disturbing Records* and includes two subthemes: the nature of disturbing material; and the consequences of exposure. A second set of themes surrounds *Empathic Engagement* including: personal connections with the material; interactions with donors; connections with community researchers; and intersections with the archivist’s sense of social justice. The final set of themes *Supports and Strategies* has two subthemes: reaching out to others; and individual emotion management.

### Disturbing records

#### Nature of disturbing material

The term “traumatic collections” has been defined as encompassing the “purposeful gathering of materials that seek to include records of disruptive, violent histories; efforts to document these events…and/or the subsequent activities that engage in truth telling, justice, and/or reconciliation” (Nathan et al. 2015, p94). Many of the collections overseen by archivists in our study indeed met this definition. This included records focussing on human tragedies such as missing and murdered Indigenous women and girls; residential schools for Indigenous children; unmarked graves of Indigenous children; sexual abuse perpetrated by the clergy; internment of Japanese Canadians; and the Holocaust. Archivists in our study described these records as ones that “You couldn't help but have an emotional response to”. For instance, records documenting residential schools and unmarked graves, included: “photographs of the schools of what they were like and people”; “a sick book [of] all the children who had ever been sick…what they died of”; details of a child who died by starvation; and other “human rights violations by so-called men of the cloth, religious people that are performing these acts.” Another archivist, in “supporting investigators”, had been immersed in records related to “criminal charges [of sexual violence] filed against the Archdiocese”. Archivists also described engagement with records of the Holocaust. For example, one archivist remarked:

[T]here was a collection that started off as postcards from the early 1940s and as I say, the last half of this collection were entirely still photographs taken when the soldiers liberated Dachau…To actually see the photos of these emaciated bodies stacked like cordwood...

In addition to the inherently horrifying nature of the content, the form and quantity of the records contributed to their distressing nature, as indicated by the statement above. The Truth and Reconciliation Commission (TRC) archives also exemplify this element. TRC records include not only administrative records and photographs, but also site visits, collection of DNA from exhumations, and videos of victim testimony. One archivist shared:

Lots of elders cautioned me about the potential harm that could be done by digging up remains for DNA sampling…it was a profoundly disturbing investigation...And when you view those statements, we had one poor videographer... We had 7000 witness statements and his job was basically to edit them.

The experiences of people in this study, and those of other published studies (Sloan et al. 2019) suggest archivists can experience distressing emotional responses when working with a range of archival material. For instance, archivists described collections that were compiled as part of the regular course of business but nevertheless contain material that they found difficult to process:

[A] ircraft accident reports… have some pretty graphic images in them if an autopsy was done or even of a crash site... Those were pretty shocking to me when I first saw them... What I think about government fonds, I don't think about things like that being in them, I think about it being boring paperwork.

Survey research by Sloan and colleagues highlights the difficulty in defining a traumatic record. That is, some seemingly innocuous records evoke a traumatic response, or alternatively a response that is “unsettling, not necessarily traumatic” (Sloan et al. 2019). As noted by one archivist, this may include records of a more general nature that include some elements of spousal violence, sexual assault and misconduct, or the death of a loved one. Another archivist described the “jarring” nature of records chronicling previous approaches to psychiatric treatment:

Largely the records that were represented in the collection documented the life and work of the people at the hospital… it's kind of hard to wrestle with the fact that a lot of people who did work there believed that they were doing the best thing for the patients there…It was shocking to come across …images of the machinery or implements that were used... We'd be going through a series of photographs of smiling nurses and then there'd be just some really grotesque looking medical equipment.

#### The consequences of exposure to disturbing material

Archivists described a wide range of responses arising from exposure to disturbing material. Some focussed on the element of surprise arising from one element of a record and the way this provoked an immediate response–experienced as a “roller coaster” ride, being “hit in the face”, “a blow to the head, or “it kind of takes the air out of my balloon that day, sometimes. I just feel kind of deflated”. Another described “feeling flushed, so I guess an increase in blood pressure, intense emotion in my chest, anger, sadness, can often lead to me crying or choking up, even tearing up”.

One archivist explained:

[S]ometimes the traumatic aspects could come up in an unexpected way. So, there might be one diary in somebody's papers that has sensitive information in it, that's gonna be triggering or traumatic for somebody. So for me, it's not necessarily the big obvious things... The emotional part comes with the smaller stuff that you may not even be thinking of or may not be recognizing as being triggering or traumatic.

Often these sudden exposures involved visual records such as photos or videos that “sticks with me”. One archivist explained:

The photograph gives it to you like straight and unfiltered. So there's that type of difference between the written word and images… A photograph of something disturbing, like war photography, it has an immediate visceral response because you're not being protected at all...They have an impact and sort of I think it's very immediate.

Other records that evoke emotional responses are more encompassing, involving prolonged and multiple exposures, “Just working in that traumatic context, day in, day out… Working 8 h a day all on my own”. One archivist described this experience as a “slow burn”, another indicated “It was like the record seeped or steeped into me”.

At times archivists in this study described significant mental health impacts of exposure to records including symptoms of post-traumatic stress disorder and depression such as: nightmares; heighted arousal and generalized fears; impaired concentration “like seeing everything through a thick pane of glass”; impaired problem solving; and substance use. Some described negative impacts on their relationship with family. For instance, “my patience was short with my kids, and I felt like I wasn't able to prioritize—all of the little things that you need to be kind of managing when you have young children, to just even get them out of the door, I wasn't able to kind of do that”. Another archivist elaborated:

I was having a really hard time leaving anything at work...I was carrying everything home, I couldn't sleep, I was really down, and so I began to go see someone like a therapist and just to work through this, and she referred to it as vicarious trauma, but she also referred to it as a kind of... It was a social work term, she said like a kind of burnout. Compassion fatigue, is that what it's called?

For others, the response was contained to work life: “Honestly, I don't think it has impacted my personal life in any way that I can remember…[P]rofessionally, I think it was more trying to keep it together in the workplace.

A prominent theme regarding the impact of exposure to certain records was a profound sense of “anger, sadness, feelings of despair”- labelled by an archivist as “vicarious grief”. One archivist reported:

I feel like my brain actually slows down in trying to process what I'm reading or what I'm seeing, and the emotion that comes up is something I've been trying to figure out, because it feels like grief...And maybe it's guilt too…that I get to live here and have this relatively, you know, very privileged life and it's built on just horror.

Another archivist remarked,

I feel like they're incredibly heartbreaking…[they] weigh on my heart and in my head. And they definitely guided those emotions of whether it was awe, or if it was sadness, or grief, or disappointment even.

The pervasive sadness arising from exposure to human suffering at the hands of others, affected the world views of archivists. One noted, “it was despair in how shitty human beings are…how do you reconcile that huge level of monstrosity and evilness in the world”. For some archivists this contributed to sense of powerlessness, related to the historic nature of the violence inflicted on others and the inability to stop the violence from occurring “feeling powerless because it's already done and there's not an outlet for making it better…I think there was a lot of struggle with, why am I doing this work? What is the outcome?” Also contributing to the guilt was a sense that these emotional reactions were not justified as described by two archivists.

I think that it's hard to sort out what exactly you're grieving. The kids on these lists, they aren't my family, so I don't think that my grief is the same as the folks who are surviving in those families.

[I]t felt difficult to feel like I could have my feelings because the person involved was the true recipient of the trauma in that situation, and I didn't want to make it about me…and so it's hard to find a place to have those feelings without feeling like I'm usurping where the attention needs to go.

On the other hand, archivists wanted to ensure that we were also aware of the positive emotions that can arise from records: “I think it's also worth perhaps noting and hopefully it's okay to note that there are funny pictures and there are joyful pictures…and that helps to balance the negative.” Indeed, as reported in previous research (Aton et al. in review), most archivists described profound satisfaction with the work, arising from the sense that they were contributing to social change, and were helping others find truth and perhaps resolution, at times easing the suffering of individuals. Words used to describe the rewards of the work included: “joyful”, “exciting”, and “inspiring”. They also described the personally transformative nature of the work, and the opportunity to grow when faced with adversity. “There’s always more to learn” said one archivist. Another archivist noted, “It made me a better professional, because I think I am more empathic, and I realized that it is not just a box a paper. This is someone’s life you are looking at”.

In summary, exposure to “traumatic records” and other records containing human injustices, violence and suffering, can lead to a wide range of emotional responses in archivists. At times the responses are immediate arising from one image or event, at other times they are the result of prolonged exposure to records with traumatic potentialities (Sexton 2019). For some archivists the emotional response pervades their work and personal lives, for others it is more limited. One archivist aptly summarized our findings by stating “You also never know what people's personal situations are and what is going to trigger one individual may not trigger another one. So, we also need to be cognizant of that”. The next set of themes surrounding empathic engagement begins to inform us about what might potentially contribute to variable responses in archivists.

### Empathic engagement

While certain types of records were ones that “you couldn't help but have an emotional response to”, in other cases responses were much more individual. In exploring this with archivists in our study, themes arose around feeling a personal connection with material that resonated with something in the archivist’s own life or history; the nature of the relationship with donors and community researchers; and the way the material contained in the records intersected with the archivist’s own sense of social justice.

#### Connection to materials

Many archivists in this study described a profound sense of personal connection with materials that they experienced as disturbing. This connection took several forms. For some archivists the material evoked memories of a previous personal trauma. For instance, one archivist shared that “I hadn't experienced the same kind of abuse that I was reading about, but the abuse that I had experienced was similar enough that it didn't take much to find similarities and kind of have those feelings resurfacing…the re-opening of trauma”. Another archivist described the acquisition of a fonds related to a disease which had caused the death of a member of her family. She said, “because of that personal connection that I had with that disease, that really had an impact on me emotionally…I was definitely traumatized”. Individuals for whom the records re-awakened a previous trauma described a sense of difficulty separating their own experience from that of people in the records. One archivist noted, “Struggling to separate, this was what my experience is, this is what someone else experienced, and trying to stay objective” and another archivist stated, “I didn’t know how to separate myself professionally from the papers”.

Some individuals described the way the records connected with their family history. This included family histories related to the Holocaust. For one archivist, “[G]enocides and intergenerational trauma were things that I was familiar with walking into archives”; family histories of residential school attendance; and family histories of psychiatric care. Others shared family histories of residential school attendance or family histories of psychiatric illness.

I discovered my mom's history, which had always been a mystery to me… Despite how many times we asked, she would never discuss it, period. And she'd always told us that she grew up in a convent, which was, now having done a lot of this research in residential schools, was a very classic way of disguising the fact that people went to residential schools.

At times individuals known to the archivist or their family members appeared in the records.

[T]hat archive is the story of my family, and like I have literally found pictures of myself, like as a kid in there… I will never have a relationship to another collection more intimately than that one…There becomes this blend between like the material that you're looking at and yourself.

At other times, similarities between experiences documented in the records and the archivist’s family and life increased the impact of engaging with certain types of records and made it “hard to leave at work. I had a hard time separating from that one”. One archivist noted “I think after I had children, I did certainly view a lot of these records from that lens especially the apprehensions of children”. Other archivists who were parents identified records related to children. For example, one archivist stated, “films of children who died, who didn’t have the fortune of living very long, that was difficult” and another noted,

There are some records that indicate deaths of students that happened at this particular institution. And [my therapist] made this connection that part of my anxiety around sending my kid back to school might also be feeding off of the work that I've been doing with these school records having to do with students dying.

In other cases, archivists developed a deep connection with the records through engaging for a prolonged period with intimate materials from someone’s life. “[B]eing privy to part of people’s lives, that they never necessarily intended that other people would be privy to or that it was a private correspondence that just ended up with us”. The following statements from two archivists in our study exemplify that sentiment:

I think there's an intimacy in having an insight into someone's life based on their archives… you're emotionally invested in the person, whether they're alive or not…whether for good for bad or you along for the ride.

[There is] a level of intimacy, with that record functioning as this sort of emotional vessel… It felt like I was entangled with the records themselves…I think that humans and records are entangled with each other…[T]hey leave their mark upon us and we leave our marks upon them, and it's kind of inextricable.

#### Connection with donors

A second form of connecting or engaging involved interactions with individuals donating archives. As noted by one archivist “even during that physical transfer of records, that day of, they're very emotional… It's like a part of their family is leaving them”. Another reflected:

[I]n a community archive it's usually because someone has died or they're making like a huge life transition. So, they're feeling emotional and they want to bring this to you and endow you with their emotions. In some way you're taking on what they feel… donations are often an emotional performance.

As a result, the archivist serves as an emotional support or emotional proxy for the donor as one archivist stated, “having to witness her grief, and to create the space to support and just be there to listen and hear the stories”. The archivist further observed “those lines of donor-archivist, institution and person, sort of have to get blurred a little bit” a sentiment earlier expressed in the literature (Cifor and Gilliland 2016). Jennifer Douglas and Alexandra Alisauskas (2021) similarly discuss the intensely personal nature of records for individuals experiencing loss of a child. They suggested that records contributed to “grief work”, validating that a life or experience occurred, and presenting a way surviving family members could express love (Douglas and Alisauskas 2021). Further, as Geoff Wexler and Linda Long suggest, archivists serve as “guardians of a personal legacy”(Wexler and Long 2009, p485).

#### Connections with researchers

The community-based researchers described by archivists in this study were sometimes victims/survivors of the trauma contained in the records. “I guess you might look at these records and see that it's a listing of names, but for folks that I saw in the room, it was often a validation of their experience and it was a way of proving that something had happened to them, so it felt a lot more profound than just a list of names”. Michelle Caswell, Marika Cifor and Mario Ramirez have described this as “to suddenly discover yourself existing” (2016, p56). In one case, the researcher was the perpetrator of the violence. “I was tasked with supervising those file reviews, and sometimes I knew exactly what was in that file and being alone in a room with someone that I knew what was in there [described in the records] was really, really tough”. Other researchers were seeking to understand their own family histories through archival work. An archivist shared that:

“[I]n giving her access to this material, not only was I giving her access to this woman who had been lost to history, but I was helping her fulfill her trauma and having lost her husband... And I felt very like, intimidated and worried about what to say.

As a consequence of interacting with individuals who have experienced loss or trauma, archivists in this study described a sense of responsibility to the community researcher, what Lowry has previously described as an “affective responsibility” (Lowry 2019). One archivist described the impact of working with researchers:

I would say 95% of the time that I was working with a researcher, they were disclosing abuses that happened to them, different traumas, addictions that they were working through, precarious housing, abuse that they were leaving, and I didn't have the tools to help them. So, I think there was that feeling of helplessness, and also just not knowing how to...I felt a responsibility.

Archivists in the study also described the obligations they felt to protect community researchers from the traumatic information contained in records:

I try to give as much context…I try to slowly broach the subject because often I don't know what they know, so asking them some questions, a bit more about this individual or so I can sort of gauge what kind of information that they already have… I don't feel the need to give them that information that's been unsolicited.

Some also felt apologetic or responsible because of the manner in which the records were kept or what they contain. This included records with “a lot of racism and derogatory slang”; and “records that weren't kept or weren't kept well… sloppy handwriting that you can barely read or the records that have gone missing or were maybe never deposited”. One archivist described her concerns with the way material was described:

[S]ometimes I feel kind of helpless because I didn't write this description and neither did my colleague, but I have no power to change this…And [the language] could be re-victimizing and traumatic for people just to do a search, they're not even looking at the document yet.

This personal aspect of the work was at times unexpected. “I think my expectations were that, there would be maybe more of a delineation between myself and the researchers…I expected a little bit more…boundary”. Another archivist explained “[T]here's this whole feeling aspect. It doesn't mesh with it being the science, right, to sort of position it is also an empathetic field. So nobody positioned it to me like that, it was positioned as a science”. Another archivist stated, “I had no idea signing up that it would be this hard”. Similar to previous findings (Sloan et al. 2019), a large proportion of archivists in this study indicated that they had no training that specifically addressed working with traumatized individuals or individuals expressing emotional distress.

Archivists in this study described different ways in which they managed the deeply personal requests of researchers. Some were personally connected to the community and used these connections to support researchers: “And I found I really connected with people. I have a complicated family history, and so I shared a lot of similar experiences in my own personal life when I was growing up, and so I think that that really helped form that connection with a lot of those researchers”. Others, however, described ways in which they attempted to separate their own experience from that of the community researcher. For example, one archivist said, “Trying to help people as best I can without being affected by their personal stories”. Another shared, “Oftentimes, it leads to sort of assessing a lot of their emotions and trying to counter mine, and… I don't want to say hold back, but to refrain from focusing on my emotions, and instead focusing on the needs of the researcher or the community member”.

#### Commitment to people/social justice

A final theme that arose with respect to engagement with the records is the way the work and what the records document, intersects with the archivist’s sense of justice. This is evidenced in what researchers have referred to as a reverence for the records or a sacred mission (Wexler and Long 2009; Douglas et al. 2019; Aton et al. in review). One archivist in our study indicated “I feel like there's a lot of responsibility to work responsibly with those records and for the communities that those records are about and taken from”. Another archivist remarked “I feel a huge responsibility to help, I work in public service. I know people have been mistreated… They are not necessarily assisted in the ways that they need to be assisted”. For one archivist this sense of responsibility resulted in a wish to protect the records from media interest or from others with voyeuristic intentions.

[T]he key motivating factor that we did not want to release any records at any point if they even had the slimmest chance of identifying someone who could have been a potential victim.

Another aspect was connecting individuals and communities with records that were rightfully theirs and exercising what Henria Aton, Wendy Duff and Megan Shields referred to as “activist potentialities” (Aton et al. in review). Two archivists described their sense of responsibility in the following ways:

If we want to be a decolonizing archive, we have to have those intimate relationships with communities to make them understand that these are their records and we're just stewards for them, and they should be guiding us at the end of the day.

I think that’s a big part of my work…looking at issues related to community engagement, and to giving over what power we have to other communities, and to really being open to letting our experiences in speaking with other communities affect and change the work that we’re doing.

In summary, as noted previously by Aton, Duff and Shields (in review) archivists in this study described a commitment to people rather than to records. This included people who donated records, people who sought answers to their own personal life questions, and people whose stories were documented in the records. Working with records with traumatic potentialities (Sexton 2019) engendered a deep sense of responsibility and a desire to use the power inherent in records. These commitments are aligned with the aims of radical empathy (Caswell and Cifor 2016) through what has been termed “archival activation” (Tai et al. 2019). Further, archivists expressed a desire and commitment to learn from the relationships with donors and community researchers and use this new knowledge to transform their practice (Watts 2017).

### Strategies/ supports

As noted earlier, many archivists in this study identified that they were unprepared for the emotional intensity of working with troubling archives. “No one tells you this and when you're studying it and when you're in archive school, when you're doing your MLIS, but like, people are going to cry” said one archivist while another stated, “It was mind-blowing to me that we didn’t have any training on how to deal with emotions in the reading room”. Further, many noted that the organizations in which they work did not acknowledge or support archivists who experienced emotional distress as a result of working with donors/community researchers or distressing materials. As a result, archivists in this study developed their own strategies. These strategies fell into two broad categories: reaching out to others; and personal strategies for emotion management including cognitive reframing and distraction.

#### Reaching out to others

Several archivists identified individuals in their own organization that supported them as they dealt with challenging emotions, a process that allowed for a degree of emotional release. One archivist noted “An elder who was here all the time, and she was always available to talk”. Another indicated “encountering a trauma that is community-specific almost provides a pre-made support system…if I had gone upstairs and burst into tears in front of my executive director and said I’m sorry I can’t work today [and explained what I found], he would have understood”. Two other archivists provided example. “I was lucky to have co-workers who a felt very comfortable talking to when things were difficult, and I could tell that they were also struggling with some of the material”; “I would definitely say our group of people, we're really like family. I would say that's how we've kind of grown together”. Others spoke about taking advantage of personal networks for instance “interact[ing] with my friends and family”.

Many archivists also indicated that they had personal therapists with whom they shared their struggles and who helped them develop personal strategies for managing emotional distress. Common themes included understanding the way the records intersected with the archivist’s personal experiences, and “we worked on a lot of cognitive behavioral strategies to separate [my emotions from the experience of those in the archives]”.

#### Personal emotion management

In addition to seeking support from others, archivists in this study described several ways in which they managed the emotional impact of working with challenging records. One approach was to create temporal distance, limiting personal exposure. Comments to this end included strategies to vary the work undertaken or take breaks. For example, “We need to switch up tasks…I’ve learned that is a good tactic for me…take breaks”; “Leaving difficult material to later “so they might sit unprocessed for a bit”; “Go for a walk around the block, go for a coffee, do something that does not engage with this right now”; and “I find running really helps”.

A second strategy involved setting boundaries between themselves and those needing assistance and setting boundaries between work and home. For instance, one archivist noted: “So, I think I just try to share as much as I can and be as available as I can, but there's also a boundary too, because I'm not their personal researcher. I work in reference”. Other archivists described clear separations between work and home life: “I wouldn’t do any work outside of my regular hours, I wouldn’t read any upsetting news about these topics” and “I definitely do try… I try to leave work at work.”

Finally, archivists described creating cognitive distance from the exposure. For instance, archivists spoke about deliberate strategies to compartmentalize. One archivist stated, “I might just try to compartmentalize my feelings and say, these personal feelings are over here, this is my work” and another remarked “I am good at compartmentalizing. Professionally I put things in boxes and then personally, I also think I put things in boxes”. When this ability to compartmentalize failed, emotional responses began to create ongoing distress and intrude on work and home life. One archivist explained: “I was becoming more and more… Not emotionally invested, I've always been emotionally invested… but I couldn't compartmentalize it anymore”.

Several archivists described explicitly differentiating their experiences from those described in the records or those experienced by donors and community researchers, in what we might understand to be cognitive empathy (Rogers 1957):

It's okay for us to feel strong emotions working with these records and finding ways to deal with that appropriately. And what I mean is not taking on all of the grief that comes up or taking on that grief as our own, but being able to separate it a little bit and compartmentalize it, and realize that I can feel sad about what I'm seeing, but this isn't necessarily the same sad that the community might feel when they come in.

That's not the same as being a survivor of trauma. So I just think it's important to remember our position as archivists and what trauma really -- really can mean or means...The records are records. They will mean different things to different people, but they're not our records… So I mean, I think it's healthy to just keep that into perspective.

## Discussion

This qualitative research project confirms and elaborates on scholarship of others (Sloan et al. 2019; Lowry 2019; Douglas et al. 2019; Aton et al. in review; Wright and Laurent 2021) regarding the potential impact on archivists of working with records involving human suffering and with donors and community researchers who are themselves a party to that suffering. What has been described as “emotional disturbances” caused by being placed within “landscapes of human suffering” (Gregory et al. 1997, pp297,300). Reactions experienced by archivists in the current study included shock and horror, intrusive thoughts, profound senses of anger, sadness and at times despair, and ultimately at times disrupted functioning in personal and occupational spheres.

As noted by an archivist in our study, some types of encounters are sufficiently horrifying that may provoke a reaction in anyone. These might be considered “traumatic collections” as defined by Nathan and colleagues (Nathan et al. 2015). However, as noted by other scholars (Sloan et al. 2019), the reactions to other types of collections were highly variable and personalized. To this end, we view archivists as non-neutral interlocutors who bring their own backgrounds and emotions to their work with records, thereby influencing the degree to which they are affected by the emotions contained within records themselves (Tai et al. 2019). We suggest that these backgrounds influence the nature of empathic engagement that archivists have with records, donors and community researchers and that the nature of empathic engagement contributes to the types of emotional responses experienced.

Empathy has long been conceptualized to include two elements: a vicarious emotional process (Hume 1777; Keefe 1976) in which the individual feels the pain and suffering of another “as if” they experienced it themselves (Damasio 2001); and a cognitive process (Kant 1788; Rogers 1957) in which the individual perceives and understands the view of another as if one were that person but without ever losing the “as if” condition (Rogers 1957). These elements of empathy have recently been confirmed as involving differing neuro-processes resulting in varying physiological and emotional reactions (Eres et al. 2015; Fan et al. 2011). In recent archival scholarship, the concept of radical empathy (Caswell and Cifor 2016) has been helpfully introduced as a means for advancing the great potential for archives to provoke change in the aid of social justice (Duff et al. 2013). However, within this concept, there remains recognition of the “grave danger for archives and archivists” (Caswell and Cifor 2016, p32) if the experiences of others are appropriated.

In this study, archivists described experiences in which records contained their own personal life histories and the life histories of their family members, or where they saw parallels between their experiences and the experiences of others including subjects of the records, donors, and community researchers. In these cases, some archivists described “re-opening of trauma” and a difficulty separating their own experience from those of individuals in the archives. Similarly, some archivists described deep emotional connections with donors who were experiencing grief or community researchers who were exploring their own histories of trauma in which “those lines of donor-archivist, institution and person, sort of have to get blurred a little bit” as one archivist remarked. We might understand these experiences to arise from emotional empathy.

On the other hand, archivists’ descriptions of strategies to manage their emotional states were more akin to notions of cognitive empathy. That is, they sought ways to separate themselves from the records temporally by taking breaks and engaging with others. They “compartmentalized” their feelings and established boundaries between themselves and community researchers, and by leaving the work behind when they returned home. Importantly, while experiencing emotional responses to the records and to the people who experienced trauma, they consciously differentiated their experiences from those of others, maintaining “perspective”.

We would argue that engaging in cognitive empathy is not antithetical to feminist ethics of care and a survivor-centric approach to archival work (Caswell 2014). Indeed, archivists in our study described a deep commitment to social justice and deliberate actions to participate in a process of empowering communities to use records to redress historical atrocities and wrongs (Duff et al. 2013). Thus, following the long tradition arising from other fields, archivists can embrace their “affective responsibilities to other parties” and use their knowledge and access to resources to “privilege the subjects of records” in the aid of social justice. But they can do this through understanding radical empathy as *“*the ability to understand and appreciate another person’s feelings, experience, etc.” (Sevenhuijsen 2003, pp24-25) without the necessity to meld their experiences with those of others (Koss-Chinoino 2006).

That said, archivists in our current study, like those of previous studies (Aton et al. in review; Sloan et al. 2019) indicated that they felt unprepared for the work that they were required to undertake when working with individuals whose lives intersected with challenging archives – either traumatic archives (Nathan et al. 2015) or those with traumatic potentialities (Sexton 2019). Thus, a new set of responsibilities arises from this work. The first is the responsibility of institutions of higher learning who are educating future archivists to: 1) provide opportunities to develop the understanding and skills to work with individuals affected by trauma; and 2) build strategies to enhance coping capacity of archivists and skills to identify when challenges are such that additional assistance is necessary. The second is the responsibility of those who employ archivists to create a culture that acknowledges the interpersonal challenges of the work and provides supports for archivists who are shouldering these challenges. To this end the Australian Society of Archivists has prepared a useful resource for coping with vicarious trauma (Laurent & Wright, 2020). Finally, archivists themselves hold responsibility to undertake their professional obligations to unleash the transformative potentials of records and support those affected by the traumatic events captured in the records, but to do so in a manner that does not compromise their own emotional safety and well-being, and continues to attend to their own needs and those of their colleagues.
